# Effects of analogue insulin in multiple daily injection therapy of type 2 diabetes on postprandial glucose control and cardiac function compared to human insulin: a randomized controlled long-term study

**DOI:** 10.1186/s12933-015-0320-2

**Published:** 2016-01-16

**Authors:** Helene von Bibra, Thorsten Siegmund, Iris Kingreen, Markus Riemer, Tibor Schuster, Petra-Maria Schumm-Draeger

**Affiliations:** Clinic for Endocrinology, Diabetes and Vascular Medicine, Klinikum Bogenhausen, Städt. Klinikum München GmbH, Munich, Germany; Institute for Statistics and Epidemiology in Medicine of the Technische Universität, Munich, Germany

**Keywords:** Analogue insulins, Human insulin, Postprandial glucose, Metabolic control, Diastolic cardiac function, Insulin resistance, Type 2 diabetes, Diastolic dysfunction

## Abstract

**Background:**

The prevention of cardiovascular disease, including diastolic cardiac dysfunction with its high prevalence and ominous prognosis, is a therapeutic challenge for patients with type 2 diabetes. Both short and long-acting insulin analogues (AI) have been shown to reduce glucose variability and provide potential benefit for cardiovascular disease although the effects on cardiac function have not yet been evaluated. This long-term, prospective, randomized controlled trial in patients with type 2 diabetes (T2D) tested the hypothesis that a multiple daily injection regimen (MDI) with AI improves postmeal glucose excursions in comparison to human insulin (HI) and that the effects of AI improve diastolic cardiac function.

**Methods:**

For 36 months, MDI treatment in 109 T2D patients was adapted every 3 months (targets: fasting glucose ≤ 110 mg/dl, postmeal glucose ≤ 150 mg/dl) in both groups: AI (insulin detemir and insulin aspart, n = 61) and HI (NPH-insulin and regular HI, n = 48). Diastolic cardiac function (myocardial velocity E’ using tissue Doppler imaging and the mitral inflow ratio E/A) and vascular function were assessed before and 2 h after a standardized breakfast (48 g carbohydrates). At baseline, both groups were comparable with regards to demographic, cardiac and metabolic data. Analysis of data included traditional statistics as well as the use of a multiple imputation technique shown in brackets [ ].

**Results:**

At 36 months, the primary endpoint, postmeal glucose, decreased by 20 ± 62 mg/dl, p = 0.038 [p = 0.021] with AI and increased insignificantly with HI (inter-group p = 0.032 [p = 0.047]) to postmeal glucose levels of 161 ± 39 with AI vs. 195 ± 54 mg/dl with HI (inter-group p = 0.002 [p = 0.010]) whereas the levels of fasting glucose and HbA1c were comparable. With AI, postmeal E’ improved by 0.6 ± 1.4 cm/s, p = 0.009 [p = 0.002] and fasting E’ by 0.4 ± 1.4 cm/s, p = 0.069 [p = 0.013], however, E’ remained unchanged with HI. These changes were consistent with those of the traditional parameter E/A.

**Conclusions:**

MDI with AI results in better postmeal glucose control compared to HI. The treatment with AI is associated with improved diastolic cardiac function.

ClinicalTrials.gov (NTC00747409)

## Background

Cardiovascular disease including heart failure is the leading cause for morbidity and mortality in people with diabetes mellitus making adequate therapy mandatory [1]. Diastolic cardiac function is already impaired in the pre-diabetic phase (impaired glucose tolerance) and brings with it an increased risk of heart failure [2, 3]. Based on the association of cardiac dysfunction with fasting and postmeal metabolic control [4, 5], diagnosis and monitoring of sub-clinically impaired cardiac function may be valuable for monitoring therapeutic efficacy [6]. As well in the pre-diabetic phase, the prevalence of myocardial infarction is alarming [7]. As consequently suggested, the improvement of postprandial metabolism, that is a reduction of glucose excursions, should be made a cornerstone in metabolic control for the prevention of cardiovascular disease [8–10], thereby shifting the focus from previous landmark studies related to the HbA1c, an overall mean glucose value, to cardiovascular risk. Taking this risk and that of heart failure in diabetes mellitus into account, the best risk–benefit ratio exists for metformin and insulin [11]. In particular, the more recently-developed short-acting insulin analogues have shown superior control of postprandial glucose levels [12] and a reduction in cardiovascular events [13]. Likewise long-acting analogue insulin preparations with their flatter profile have shown advantages in day-to-day glucose variability compared to human insulin [14]. Consequently, the combination of short and long-acting insulin analogues (AI) in a multiple daily injection regimen (MDI) theoretically offers cumulative effects for the reduction of glucose excursions/variability.

Whether MDI with AI can improve myocardial dysfunction in patients with type 2 diabetes has not yet been assessed. As a proof of concept, this prospective, randomized, open, long-term study tested the hypothesis that MDI with AI improves postprandial glucose better than human insulin (HI). Furthermore, a beneficial effect of AI on diastolic cardiac function was to be evaluated.

## Methods

### Patients

**Fig. 1:**
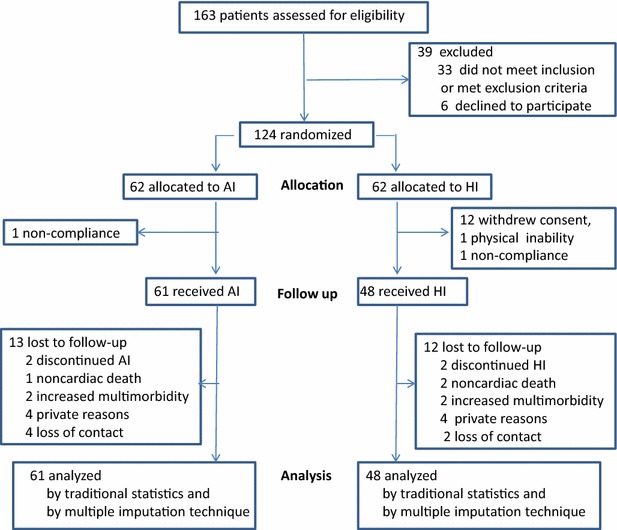
Trial profile for the long-term RCT study MDI with analogue (AI) versus human insulin (HI) in type 2 diabetes

This prospective long-term (36 months) study on the comparison of AI versus HI for MDI regimens randomly assigned 124 Caucasian subjects with insulin-treated type 2 diabetes either to treatment with analogue insulin or human insulin (Fig. 1). All patients attended the Clinic of Endocrinology, Diabetes and Vascular Medicine of the Academic Teaching Hospital Bogenhausen in Munich between 2004 and 2009. Included were patients with insulin-treated type 2 diabetes of both sexes between 35 and 85 years after having submitted their written, informed consent. Exclusion criteria were increased left ventricular diameter (>55 mm), any signs or history of heart failure, >mild grade valvular heart disease, pericardial disease, atrial fibrillation, severe diabetic neuropathy or retinopathy, creatinine >2 mg/dl and untreated thyroid dysfunction.

The study was conducted in accordance with the Declaration of Helsinki and Good Clinical Practice Guidelines. The protocol and the consent form were reviewed by the local independent ethics committee before the start of the trial.

### Randomization and masking

Using an independently-generated randomization list, patients were consecutively assigned to one of two insulin regimens after enrollment: AI (insulin aspart and insulin detemir; insulin by Novo Nordisk, Bagsvaerd, Denmark) or HI (regular insulin and NPH; insulin by Novo Nordisk, Bagsvaerd, Denmark). An open label design was used as the administration time of the meal-related insulin differed between the two insulin regimens. However, any staff involved in ultrasound investigations, blood tests or the definition of data sets for statistical analysis remained blinded until the database was locked.

### Procedures

At the initial visit, all participants were instructed on how to measure plasma glucose and how to adjust the doses of fast-acting and long-acting insulin and each received an electronic device for plasma glucose determinations (ACCU-CHEK^®^ SENSOR, Roche, Grenzach, Germany). Treatment targets were defined as ≤110 mg/dl for fasting plasma glucose and ≤150 mg/dl for postprandial plasma glucose. At clinic visits every 3 months, participants received individual recommendations by a diabetologist for adjusting insulin doses based on their self-measured plasma glucose levels in order to achieve the specified glucose targets. All patients received individualized advice concerning diet, exercise and lifestyle in accordance with the guidelines [15].

At baseline and during clinic visits at 12, 24, and 36 months, the following measurements were taken: weight, blood tests and ultrasound examinations in the fasting state prior to the morning dose of fast-acting insulin. Blood and ultrasound examinations were repeated 2 h after a standardized meal that consisted of carbohydrates of mixed glycemic index (48 g) without butter and protein [5] and administration of the morning dose of meal-related insulin (usual type and dose of insulin for this meal). Daily insulin doses, state of diabetic complications, concomitant medication and its changes were documented, as well as cardiovascular adverse events as defined by hard endpoints (myocardial infarction, stroke and cardiovascular death) and soft endpoints such as heart failure, coronary angiography, percutaneous transluminal coronary angioplasty, new/worsening angina pectoris, claudication and amputation.

The primary study endpoint was the change of postprandial plasma glucose from baseline to the end of study at 36 months.

Secondary endpoints were changes in cardiac diastolic function as well as changes in cardiovascular function and metabolic variables such as.

- diastolic myocardial function measured as myocardial velocity E’ (cm/s), transmitral inflow ratio E/A, E/E’,
- vascular function as pressure-strain elasticity modulus (kPa), blood pressure,
- metabolic control with HbA1c, fasting plasma glucose, lipid profile, serum insulin, proinsulin, body weight and
- cardiovascular risk/adverse events as mentioned above.

### Echocardiography

Patients were examined while in the left lateral decubitus position using a commercially-available system (SSD-5500, ALOKA, Tokyo, Japan) equipped with tissue Doppler imaging and a 2.5–5 MHz phased array transducer. The ECG was simultaneously recorded. Standardized cross-sectional 2-dimensional and M-mode echocardiography [16] was used to measure the short axis left ventricular dimensions in the parasternal long axis view and the longitudinal left atrial diameter in the apical view. Pulsed wave Doppler imaging of the transmitral inflow was used to assess the traditional parameters of left ventricular diastolic function: early (E) and late diastolic (A) velocities and E/A.

### Global left ventricular function by pulsed tissue Doppler

As described above, tissue Doppler imaging was performed by one experienced cardiologist [5, 17, 18]. In short, peak systolic velocity (S’), early diastolic velocity (E’) and late diastolic velocity (A’) were averaged from six basal myocardial regions (apical 4-, 3- and 2-chamber view) as a measure of global left ventricular function. Given the steep and known decline of early diastolic myocardial velocity by 1 % every year due to physiologic aging in normal non-diabetic persons, individual patients were classified as subject to dysfunction if the measured diastolic velocity E’ was lower than the age-related cut off level deemed normal as calculated by the respective regression equation [18]. Left ventricular filling pressure was calculated as E/E’.

### Vascular ultrasound

Using standardized methods at the right common carotid artery, intima media thickness was measured using a 13 MHz linear array transducer. The arterial stiffness parameters elasticity modulus ε and pulse wave velocity (PWV) were measured using a combined Doppler and echo-tracking system (SSD-5500, ALOKA, Tokyo, Japan) that provided online calculation taken from instantaneous intravascular diameter and pressure changes as previously described [5, 19]. During vascular imaging, blood pressure was measured three times at the left arm by an automated cuff sphygmomanometer and averaged.

### Laboratory

Fasting plasma glucose, serum insulin and lipid profile, glycated hemoglobin A1c (HbA1c) and high-sensitive C-reactive protein (hsCRP) were determined according to routine methods at the Department of Clinical Chemistry (Klinikum rechts der Isar, Technical University, Munich). Intact proinsulin was assessed in representative subgroups (AI n = 36 and HI n = 31) by the IKFE institute, Mainz [20].

### Statistics

Drawing from experience in an earlier study comparing analogue with human insulin therapy over the course of 1 year, a difference of 10 ± 19 mg/dl in postprandial glucose between the treatment groups [12] was expected. In order to detect this difference with a power of 80 % at a 5 % significance level, each treatment group would need to have 58 patients. For the secondary endpoint diastolic myocardial velocity, a difference of 0.6 ± 1.1 cm/sec was expected between the groups. The two-tailed T test with n = 58/group would then yield a power of 82 %. Assuming an expected drop out rate of 10 %, the unpredictability of standard deviations and the test result for the secondary endpoint diastolic myocardial velocity, ≥60 patients would be necessary for each treatment arm.

Statistical analyses were performed using the SPSS version 18.0 software package for windows (SPSS Inc, Chicago, IL). Data was expressed as mean ± standard deviation when normally distributed and otherwise as median {interquartile range}. The difference between baseline and endpoint measurements of a specific parameter was calculated as delta (Δ) and the difference between postprandial and fasting values as a postprandial excursion. Students’ T test or nonparametric tests were used for group comparisons where appropriate. The study data were analyzed in conventional statistics. To compensate for the uncertainty caused by missing values from drop-out individuals, data were additionally analyzed using the multiple imputation technique with 10 replacements at random in order [21] based on the confirmation that the missing pattern was completely at random [22]; these results are presented in brackets [ ]. Bivariate correlations were assessed using Pearson’s correlation coefficients.

### Role of the funding source

The study design, protocol and statistical plan of this investigator-initiated study were provided by both first and the last authors and were agreed upon by the study sponsor who supplied the study products. The authors alone contributed to data collection, interpretation and writing of the report and take full responsibility for its content.

## Results

There were no significant differences between the pre-study insulin regimens of the different treatment groups with regards to analogue or human insulin, basal bolus or other insulin strategies. Participants of both study groups were comparable for demographic, hemodynamic, cardiac and metabolic data including cardiovascular risk factors and concomitant medication (Table 1).

**Table 1:** Demographics and baseline data of patients

		Analogue insulin group AI (n = 61)	Human insulin group HI (n = 48)	p
Age	Years	60.4 ± 9.5	63.1 ± 10.6	0.159
Sex male	n (%)	46 (75)	31 (65)	0.218
BMI	kg/m^2^	32 ± 5	32 ± 6	0.709
Duration of diabetes	Years	10 {5–15}	9.5 {5–14}	0.580
Insulin dose MDI	IE/d	56 {37–83}	50 {36–70}	0.485
Metformin	n (%)	13 (21)	16 (33)	0.159
Hypertension	n (%)	58 (95)	42 (88)	0.153
Smoking	n (%)	17 (28)	15 (31)	0.700
Myocardial infarction	n (%)	14 (23)	8 (17)	0.417
Betablocker	n (%)	23 (38)	18 (38)	0.983
ACE-inhibitor	n (%)	37 (60)	33 (69)	0.224
Calcium channel blocker	n (%)	9 (15)	10 (21)	0.406
AT1 blocker	n (%)	15 (25)	8 (17)	0.314
Diuretics	n (%)	23 (38)	20 (42)	0.674
Statins	n (%)	28 (46)	25 (52)	0.522
Aspirin	n (%)	30 (49)	23 (48)	0.896
Glucose fasting	mg/dl	163 ± 54	163 ± 45	0.962
Glucose pp	mg/dl	183 ± 69	185 ± 50	0.870
HbA1c	%	7.3 ± 1.7	7.7 ± 1.8	0.237
Intact proinsulin^a^	pmol/l	5 {2.7–7.6}	4.8 {3.2–8.2}	0.515
Triglycerides	mg/dl	137{102–177}	117 {94–184}	0.463
Cholesterol	mg/dl	187 ± 36	188 ± 42	0.890
LDL	mg/dl	114 ± 34	113 ± 37	0.941
HDL	mg/dl	46 {38–53}	48 {39–54}	0.543
Creatinine	mg/dl	0.9 {0.8–1.08}	0.9 {0.7–1.1}	0.493
ASAT	U/l	29 {24–40}	27 {23–39}	0.862
CRP	mg/dl	0.28 {0.13–0.5}	0.3 {0.15–0.58}	0.552
S’	cm/s	7.6 ± 1.1	7.6 ± 1	0.984
E’	cm/s	7.8 ± 1.4	8.2 ± 1.7	0.168
LV end-diastolic diameter	mm	44 {42–48}	44 {41–48}	0.776
Septal thickness	mm	12 {11–14}	12 {11–13}	0.449
Mitral E/A		0.9 {0.7–1.1}	0.9 {0.8–1.1}	0.651
E/E’	mmHg	8.9 {7.5–10.9}	8.1 {7.0–9.6}	0.174
Intima media thickness	mm	0.7 ± 0.2	0.7 ± 0.2	0.714
Εlasticity modulus Ɛ	kPa	140 {113–228}	165{136–259}	0.087
Pulse wave velocity	m/s	7.3 {6.6–9.2}	8.3 {7.0–9.9}	0.151
Heart rate	bpm	71 {63–78}	69 {64–80}	0.509
Systolic blood pressure	mmHg	140 {129–152}	140{123–150}	0.712
Diastolic blood pressure	mmHg	83 ± 12	81 ± 9	0.321

Mean ± SD or median {interquartile range} respectively

*pp* postmeal, *S’* systolic myocardial velocity, *E’* early diastolic myocardial velocity, *LV* left ventricular, *E/E'* estimated LV filling pressure

^a^Data from representative subgroups (AI = 36 and HI = 31)

Of the 109 patients, 5 patients in group AI and 4 in group HI were lost to follow-up at the 24-month visit, and 8 patients in each group at the 36-month visit (Fig. 1). The resulting data were analyzed both using conventional statistics and the multiple imputation technique as shown in brackets [ ]. The mean treatment period was 32 ± 7 months.

At 36 months, the daily insulin dose per kg body weight had significantly increased: by 0.07 ± 0.28 U/kg with AI and by 0.18 ± 0.26 IU/kg with HI (inter-group significance p = 0.054). Weight had increased from baseline to month 36 with AI by 2.3 ± 7.8 kg (p = 0.050) and by 2.8 ± 7.6 kg (p = 0.038) with HI (inter-group significance p = 0.774).

At 36 months (Table 2), the primary endpoint, postprandial glucose, had decreased by 20 ± 62 mg/dl (p = 0.038) with AI but had a non-significant increase by 9 ± 53 mg/dl (p = 0.33) with HI thereby resulting in a significant difference between the groups (p = 0.032 [p = 0.047]). Simultaneously, the postmeal glucose levels (161 ± 39 with AI vs. 195 ± 54 mg/dl with HI) were relevantly and significantly different (p = 0.002 [p = 0.010]) as were the postmeal glucose excursions (15 ± 43 with AI vs. 57 ± 40 mg/dl with HI, p = 0.001 [p = 0.007]).

**Table 2:** Changes at 36 months from baseline

Parameter (dimension)	Analogue insulin group AI (n = 61)	p within-group	Human insulin group HI (n = 48)	p within-group	p inter-group
Glucose fasting (mg/dl)	−7 [−12] ± 52 [53]	0.375 [0.095]	−17 [−19] ± 42 [44]	*0.021 [0.008]*	0.336 [0.510]
Glucose pp (mg/dl)	−20 [−20] ± 62 [64]	*0.038 [0.021]*	9 [4] ± 53 [56]	0.335 [0.676]	*0.032 [0.047]*
HbA1c (%)	−0.4 [−0.6] ± 1.5 [1.7]	0.063* [0.011]*	−1.3 [−1.2] ± 1.8 [1.7]	*0.000 [0.000]*	*0.024* [0.082]
Intact proinsulin^a^ (pmol/l)	−0.9 {−2.3 to 0.8}	0.171	0.3 {−0.9 to 2.7}	0.199	*0.048*
Triglycerides (mg/dl)	0 [1] {−12 [−18] to 33 [34]}	0.496 [0.577]	11 [8] {6 [−15] to 41 [41]}	*0.016* [0.167]	0.153 [0.467]
Triglycerides pp (mg/dl)	10 [9] {−15 [−22] to 39 [45]}	0.081 [0.198]	24 [17] {−4 [−10] to 64 [55]}	*0.023 [0.040]*	0.317 [0.424]
Cholesterol (mg/dl)	10 [10] ± 49 [50]	0.161 [0.186]	6 [6] ± 43 [42]	0.437 [0.375]	0.668 [0.699]
LDL (mg/dl)	5 [3] ± 40 [43]	0.420 [0.685]	−7 [−5] ± 39 [38]	0.262 [0.408]	0.170 [0.367]
HDL (mg/dl)	1 [0] {−3 [−5] to 4 [5]}	0.636 [0.628]	2 [1] {−6 [−6] to 9 [9]}	0.493 [0.487]	0.844 [0.668]
Creatinine (mg/dl)	0 [0] {−0.10 [−0.10] to 0.10 [0.10]}	0.954 [0.710]	0.10 [0.09] {0 [−0.03] to 0.10 [0.15]}	*0.018 [0.009]*	0.054 [0.064]
ASAT U/l	−2 [−1] {−7 [−11] to 3 [6]}	0.096 [0.400]	1 [1] {−4 [−11] to 10 [11]}	0.586 [0.734]	0.126 [0.554]
hsCRP (mg/dl)	0 [−0.01] {−0.18 [−0.24] to 0.1 [0.14]}	0.468 [0.521]	0.05 [0.01] {−0.1 [−0.19] to 0.15 [0.18]}	0.307 [0.669]	0.199 [0.525]
LV enddiast. diameter (mm)	2 [2] {−2 [−2] to 6 [5]}	*0.041 [0.032]*	1 [1] {0 [−1] to 3 [3]}	0.149 [0.151]	0.387 [0.395]
Septal thickness (mm)	0 [−0.1] {−2 [−1.4] to 2 [2]}	0.948 [0.733]	0 [0] {−2 [−1.6] to 1 [1.7]}	0.718 [0.706]	0.768 [0.722]
Post. wall thickness (mm)	1 [0.8] {−1 [−0.9] to 2 [1.4]}	0.103 [0.082]	1 [0.9] {0 [−0.4] to 2 [2]}	*0.010 [0.012]*	0.388 [0.501]
S’ (cm/s)	0 [0] ± 0.8 [0.8]	0.889 [0.801]	0.1 [0.1] ± 1 [0.9]	0.504 [0.571]	0.516 [0.753]
E’ (cm/s)	0.4 [0.5] ± 1.4 [1.4]	0.069* [0.013]*	0.1 [0] ± 1.3 [1.3]	0.738 [0.853]	0.312 [0.059]
E’ pp (cm/s)	0.6 [0.6] ± 1.4 [1.4]	*0.009 [0.002]*	0.2 [0.1] ± 1.4 [1.5]	0.435 [0.430]	0.241 [0.110]
E/E’ (mmHg)	−0.1 [−0.01] {−1.52 [−1.54] to 1.54 [1.46]}	0.870 [0.657]	0.35 [0.25] {−0.9 [−1.02] to 1.99 [1.98]}	0.136 [0.182]	0.192 [0.198]
IMT (mm)	0.03 [0.05] ± 0.17 [0.18]	0.200 *[0.048]*	0.06 [0.07] ± 0.19 [0.18]	0.091* [0.018]*	0.551 [0.649]
Elasticity modulus (kPa)	15 [8] {−36 [−40] to 49 [60]}	0.363 [0.463]	30 [8] {−41 [−65] to 78 [68]}	0.313 [0.622]	0.643 [0.740]
PWV (m/s)	0.2 [0.1] {−0.9 [−0.9] to 0.9 [1.1]}	0.684 [0.494]	0.5 [0] {−1 [−1.4] to 1.7 [1.4]}	0.319 [0.622]	0.448 [0.693]
Heart rate (bpm)	−4 [−4] {−10 [−10] to 5 [4]}	0.057* [0.026]*	−4 [−4] {−7 [−7] to 3 [3]}	*0.026* [0.079]	0.920 [0.630]
Systolic BP (mmHg)	3 [7] {−9 [−9] to 20 [20]}	0.175 [0.087]	20 [17] {−5 [−4] to 29 [29]}	*0.008 [0.002]*	0.065 [0.075]
Diastolic BP (mmHg)	5 [5] ± 12 [12]	*0.005 [0.003]*	10 [11] ± 12 [12]	*0.000 [0.000]*	0.062 [0.064]

Italics p values indicate significant changes at 36 months

Mean ± SD or median {interquartile range} respectively; additional results from multiple imputation analysis in brackets [ ]

*pp* postprandial, *LV* left ventricular, *E/E’* estimated LV filling pressure, *EA* ratio of mitral inflow velocities, *IMT* intima media thickness, *PWV* pulse wave velocity, *BP* blood pressure

^a^Data from representative subgroups (A = 36 and H = 31)

Within the metabolic secondary endpoints, fasting glucose did not significantly decrease with AI but did significantly decrease with HI (Table 2) to comparable levels in both groups (150 ± 34 with AI vs. 144 ± 40 mg/dl with HI, p = 0.433). Intact proinsulin in the fasting state decreased insignificantly with AI and increased insignificantly with HI, resulting in a significant difference between these changes (p = 0.048). HbA1c decreased by 0.4 ± 1.5 %, p = 0.063 [p = 0.011] with AI and by 1.3 ± 1.8 %, p = 0.001 [p = 0.001] with HI. This larger decrease (p = 0.024 [p = 0.082]) resulted in comparable levels (6.7 ± 0.8 % with AI vs. 6.5 ± 0.7 % with HI, p = 0.486). Only with HI did fasting and postprandial serum triglyceride levels increase significantly as did serum creatinine with a trend to significance for this change between the groups (Table 2).

Within the cardiovascular secondary endpoints, there was no significant change of E’ with HI (Table 2) but postmeal E’ improved with AI by 0.6 ± 1.4 cm/s, p = 0.009 [p = 0.002] and fasting E’ as a trend in traditional statistics (by 0.4 ± 1.4 cm/s, p = 0.069) and significantly by multiple imputation analysis [0.5 ± 1.4 cm/s, p = 0.013]. Accordingly, diastolic dysfunction [12] was reduced by 22 % with AI and by 12 % with HI. The traditional parameter of diastolic function, E/A, significantly improved with AI, both in the fasting state and postmeal, and as a trend with HI (Table 3). The mitral inflow E increased significantly postmeal with AI but decreased as a trend with HI. With values close to normal, E/E’ did not change significantly. None of the changes in diastolic function parameters achieved significance between the treatment groups.

**Table 3:** Parameters of diastolic function in the fasting state and post meal (pp)

Parameter	MDI insulin regimen	At baseline	At 36 months	p
E’ (cm/s)	Analogue Human	7.8 ± 1.4 8.2 ± 1.7	8.4 ± 1.7 8.2 ± 1.7	0.069 0.738
E’ pp (cm/s)	Analogue Human	7.6 ± 1.6 8.1 ± 1.8	8.5 ± 1.6 8.2 ± 1.8	*0.009 * 0.435
E/A	Analogue Human	0.96 ± 0.3 0.93 ± 0.25	1.11 ± 0.39 1.01 ± 0.30	*0.030 * 0.061
E/A pp	Analogue Human	0.94 ± 0.30 0.92 ± 0.26	1.18 ± 0.46 1.01 ± 0.32	*0.005 * 0.055
E (cm/s)	Analogue Human	70 ± 17 70 ± 20	74 ± 19 72 ± 17	0.296 0.097
E pp (cm/s)	Analogue Human	71 ± 18 74 ± 20	76 ± 21 72 ± 15	*0.001 * 0.068
A (cm/s)	Analogue Human	76 ± 18 75 ± 21	70 ± 19 74 ± 23	0.061 0.368
A pp (cm/s)	Analogue Human	71 ± 18 74 ± 20	68 ± 18 75 ± 22	0.430 0.957
E/E’ (mmHg)	Analogue Human	8.9 {7.5–10.9} 8.1 {7.0–9.6}	8.5 {7–10.3} 8.4 {7.5–10.0}	0.870 0.136
E/E’ pp (mmHg)	Analogue Human	8.3 {7.1–10.1} 8.3 {7.3–9.9}	8.3 {7.2–10.8} 8.1 {7.4–10.8}	0.670 0.203

Italics p values indicate significant changes at 36 months

Mean ± SD or median {interquartile range} respectively

*E’* early diastolic myocardial velocity by tissue Doppler, *E* early diastolic mitral inflow velocity, *A* late diastolic mitral inflow velocity, *E/A* ratio of mitral inflow velocities, *E/E’* estimated LV filling pressure

Compared to HI, treatment with AI tended to result in smaller increases in systolic and in diastolic blood pressure (Table 2). In parallel, vascular stiffness was lower after 36 months of therapy, expressed as elasticity modulus ε (154 {105–204} vs. 182 {148–234} kPa in HI, p = 0.028 [p = 0.198].

After 36 months, there were two cardiovascular events with AI (myocardial infarctions) vs. five events with HI (one myocardial infarction, four strokes), equivalent to event-free percentages of 96 vs. 81 % (p = 0.142). Anti-hypertensive medication remained comparable between both groups.

## Discussion

This prospective, randomized, open, controlled trial showed that long-term MDI with AI in patients with type 2 diabetes resulted in better postprandial glucose control than MDI with HI. This benefit with AI was associated with improved diastolic cardiac function whereas this function remained unchanged with HI.

The improvement of the primary endpoint postmeal glucose levels following an MDI with AI regimen could be mainly attributed to the effects of the short-acting analogues. This effect has been shown in type 1 and in type 2 diabetes [12, 14, 23, 24]. The study design also provided a potentially beneficial effect from the basal analogue insulin which should result in a relative protection of the beta-cell from the flatter and longer action profile of the basal insulin [14]. An augmented beta-cell function may also be related to an improvement in insulin resistance. Our data suggest an at least relative improvement of insulin resistance by the unchanged triglycerides levels with AI versus the significant rise with HI—in spite of similar weight changes and identical nutritional recommendations in both groups.

By contrast to these specifically different metabolic effects, both treatment regimens improved long-term glucose control to a similar HbA1c level after 36 months. This similarity in long-term glucose control may result from three aspects: first, a compensation of the lower postprandial glucose levels by the higher fasting glucose levels in the analogue regimen and vice versa of the higher postprandial glucose levels by the lower fasting levels with HI. Second, a numerically higher baseline HbA1c level in the HI group (7.7 vs. 7.3 %, Table 1) allowed for a more pronounced lowering; and third, a more pronounced increase in the daily insulin dose per kg body weight with HI.

Comparative trials using the same target for MDI with different insulin types allow for minimizing potential differences in glucose control so that differences in cardiovascular risk may be assessed. In the presence of comparable fasting glucose and HbA1c levels, the results can thereby be interpreted for ultimate risk–benefit assessments including diastolic cardiac function. Improvement of diastolic dysfunction, that is the antecedent to diastolic heart failure i.e. heart failure with preserved ejection fraction (HFpEF), has been a challenging topic in the treatment of patients with type 2 diabetes. Remarkably, there is no evidence-based therapy available against HFpEF [2]. Diastolic dysfunction has high prevalence in overweight individuals with type 2 diabetes [2, 6, 18] and results in a poor prognosis and reduced quality of life because of the associated limitation of physical activities in daily life. However, it is under-diagnosed in clinical routine and lacks evidence-based treatment strategies due to problems associated with the bashful and resigned obese patients [6], with the measuring technique [6, 18] and with the understanding of the complex underlying mechanisms [25–27].

With regards to diagnostic issues stemming from the measuring technique, quantitative pulsed tissue Doppler imaging emerged 20 years ago and has since provided very good results for the sensitive measurement of myocardial velocity E’ during early diastole [26], with good feasibility even in overweight individuals at bedside, sensitivity to energy restraint, correlation to exercise capacity, prognostic value and the potential to monitor therapeutic interventions [6, 28]. Furthermore, E’ allows for the quantification of diastolic dysfunction by using the dominant influence of aging on E’ [18], which is essential for the mathematically-correct identification of risk factors and for the diagnosis of dysfunction/HFpEF. In line with earlier reports [2, 6] diastolic dysfunction was highly prevalent in the study cohort and was normalized in 22 % of the patients on AI treatment vs. 12 % of the patients on HI treatment.

In the present study design, E’ was averaged from 6 basal myocardial regions and, therefore, taken as measure of global left ventricular function. The increase of E’ with AI was mirrored by the decrease of the traditional parameter E/A in the fasting state and postmeal. Accordingly, this study demonstrated a long-term improvement in diastolic function using MDI with AI (Table 3). However, the third parameter of diastolic function, the ratio E/E’, did not change significantly with the treatment interventions due to the parallel modifications of E and E’ (Table 3). This thereby substantiated concerns in earlier reports that this estimation of LV filling pressure may be a less sensitive measure of diastolic function, especially in metabolic disease with its predominantly mild degree of diastolic dysfunction [18, 29].

It is of interest that the observed changes in diastolic function were more distinct in the postmeal state. As for the complex mechanisms and potential factors that may influence diastolic dysfunction [18, 25], this study did not demonstrate any improvement in the hemodynamic factors blood pressure or vascular stiffness. Within metabolic factors, fasting glucose and HbA1c had improved to comparable levels at 36 months. In line with recent reports [30–32], these data do not support an automatic translation of improved glycemic control into augmented cardiac function. Improvements in postprandial glucose excursions with AI were paralleled by functional amelioration, whereas the HI group demonstrated excessively high levels of postmeal glucose and its variability. These observations rather support the importance of controlling postprandial glucose and the underlying insulin resistance as therapeutic targets for improvement of cardiac function [3], at least in moderately well-controlled type 2 diabetes, in line with earlier reports [5, 18, 27, 28, 31, 33].

Pharmacological therapy with MDI or rosiglitazone and maintaining an adequate diet [5, 28, 34] have been seen to improve diastolic cardiac function in type 2 diabetes along with improved postmeal glucose. This is in agreement with the reported link between acute glucose fluctuations and activation of oxidative stress [35]. A recent study demonstrated an immediate and highly repetitive deterioration of cardiac function after a carbohydrate meal associated with increased oxidative stress in overweight and insulin-dependent patients with type 2 diabetes [36]. The reverse being also true, cardiac function has been shown to improve when adhering to diets that effectively lower weight or at least postprandial glucose levels [28, 34, 37, 38]. Small pharmacological studies have suggested positive effects on diastolic dysfunction from optimized metabolic control in patients with type 1 and type 2 diabetes [6] in particular by improving postmeal glucose control, insulin resistance and reducing oxidative stress [5, 37]. By using a practical combination of therapeutic strategies in the form of adapted anti-diabetic medication and adhering to effective nutrition [28, 39] in line with the most recent dietary treatment guidelines [40], improved postmeal glucose control appears to be a promising target for the improvement of diastolic dysfunction.

The underlying biochemical mechanisms are complex and not yet fully understood. Important metabolic mechanisms imply that a reduction of insulin resistance [28, 31, 37], oxidative stress and augmented NO bioavailability [28, 41, 42] enhance both cardiac efficiency i.e. intracardiocyte energy production [43, 44] and endothelial function that translates into augmented myocardial perfusion and function. Indeed, small clinical studies in type 2 diabetes have demonstrated that improved metabolic control may enhance myocardial perfusion and function [4] and that short-acting analogue insulin normalizes myocardial perfusion in the postmeal state by 35 % when compared to being left untreated [45]. When looked at together, these observations and the present study suggest that analogue insulin may result in optimized postmeal regulation of myocardial perfusion and hence diastolic function in type 2 diabetes. Because of its significant clinical relevance, further studies which explore the major mechanisms of postmeal (dys-) regulation of myocardial perfusion in diabetic individuals would be highly welcome.

It is conceivable that other more underlying mechanisms for diastolic dysfunction relate to insulin resistance [27, 33], as also suggested from more recent studies [3, 18, 32, 43]. For the present insulin-dependent patients with diabetes, however, the assessment of insulin resistance cannot be based on the HOMA-IR, but rather on surrogate parameters such as the daily insulin dose. The insulin dose per kg body weight increased by 0.07 U/kg with AI vs. 0.19 IU/kg (p = 0.054) with HI. Other surrogate parameters are the triglyceride levels which remained unchanged with AI but increased with HI therapy both fasting and postmeal. These data indicate not only the degree of impact insulin resistance has as a therapeutic target in type 2 diabetes. It also suggests selecting analogue over human MDI, possibly due to the amelioration of postmeal blood glucose excursions with the consequent reduction of acute glucose toxicity. In this respect, an insulin pump therapy using short-acting insulin analogues may be an even more interesting model for patients with type 2 diabetes: Glucose variability is further reduced compared to a MDI with short and long-acting insulin analogues [46].

Much more knowledge is needed with regards to the effects and reversibility of insulin resistance for optimizing fat and glucose dysmetabolism—especially postmeal [47]. Clinical studies are needed which take into account well-defined metabolic and lifestyle characteristics so that treatment strategies can evolve that make use of the inherent reversibility potential from postprandial glucose and fat dysmetabolism. This affects both the evaluation of traditional anti-diabetic medication and the most recent therapeutic developments [48, 49].

In spite of the improvements in postprandial metabolic control and diastolic cardiac function by AI, cardiovascular events were not significantly different between both treatment groups. This is due to the low patient number (n = 109) event rate (group A: 2 events; group H: 5 events) and observation period in comparison to the Steno study (n = 180; observation period 13.3 years) or the UKPD study (n = 5102; 20 years [10, 50]. Given the prognostic value of diastolic cardiac function, this study promotes the use of more actual measures of positive clinical outcome, such as diastolic cardiac function, to assess therapeutic efficiency in the treatment of type 2 diabetes.

## Limitations

Several study limitations need to be addressed. One of these is the considerable number of patients who withdrew their study participation consent or were lost during follow up. However, the results nevertheless demonstrated a significant outcome for the primary endpoint indicating that the study had indeed remained sufficiently powered. In order to evaluate the problem of missing data, an additional statistical technique, the multiple imputation technique [21, 22], was applied that was specifically developed for analyzing data sets with missing entries. The similarity of these results to the results from traditional statistical analysis supports the relevance of the study data and their statistical meaning in spite of the drop-out rate.

The larger number of patients in the human insulin group withdrawing their participation consent before the onset of therapy may have introduced a retrospective randomization bias. However, patients were free to withdraw participation at any time during the study and it seems a number had signed consent only in the hope to be randomized to a more modern and more expensive analogue insulin. These patients, in particular, were very motivated to apply optimal anti-diabetic care so that their absence may have contributed to the initially slightly higher HbA1c level in the human insulin group.

The study design did not allow for diagnosing and assessing insulin resistance by HOMA-IR in the insulin-dependent patients with type 2 diabetes. An assessment by clamp technique would have been beyond the scope of this study. Altogether, this diagnostic problem simply reflects an unsolved but important issue for the tailoring of optimal metabolic control in diabetology and should be further evaluated with respect to the specific effects of dietary and/or exercise intervention in support of pharmaceutical treatment in different stages of insulin resistance.

The improvement of diastolic cardiac function with AI compared to the unchanged function with HI did not result in inter-group significance in the fasting state (p = 0.312 [p = 0.059] or postmeal (p = 0.241 [p = 0.110] due to an unexpectedly high standard deviation of the change from baseline, so that the study turned out underpowered for comparative assessment of cardiac function. This limitation, however, should not detract from the clinically-relevant message that MDI with AI does improve diastolic cardiac function in moderately well-controlled patients with type 2 diabetes.

## Conclusions

This report provides evidence from a randomized controlled long-term study that multiple daily injection therapy with analogue insulin is superior for the control of postprandial glucose in comparison to human insulin. Additionally, MDI with AI is associated with improved diastolic cardiac function. This is a step forward in the treatment of diabetic patients with diastolic dysfunction who are at risk for heart failure with preserved ejection fraction. Further exploration is warranted as to the impact of postmeal glucose excursions and the underlying insulin resistance for the regulation of myocardial perfusion both with the help of lifestyle changes and anti-diabetic medication. For the benefit of the patients, it would be helpful for future studies to also include measures of actual clinical outcome, such as diastolic cardiac function, in addition to traditional cardiovascular endpoints.
